# Spatial and temporal variations relevant to tsetse control in the Bipindi focus of southern Cameroon

**DOI:** 10.1186/1756-3305-6-193

**Published:** 2013-07-01

**Authors:** Judith Tchouomene-Labou, Hugues Nana-Djeunga, Gustave Simo, Guy Roger Njitchouang, Gerard Cuny, Tazoacha Asonganyi, Flobert Njiokou

**Affiliations:** 1 Department of Animal Biology and Physiology, Parasitology and Ecology Laboratory, Faculty of Science, University of Yaounde I, P.O. Box 812, Yaounde, Cameroon; 2 Centre for Research on Filariasis and other Tropical Diseases, P.O. Box 5797, Yaounde, Cameroon; 3 Department of Biochemistry, Faculty of Science, University of Dschang, P.O. Box 67, Dschang, Cameroon; 4 UMR 177, IRD-CIRAD, CIRAD TA A-17/G, Campus International de Baillarguet, Montpellier, Cedex 5, 34398, France; 5 Faculty of Medicine and Biomedical Sciences, University of Yaounde I, P.O. Box 1364, Yaounde, Cameroon

**Keywords:** Sleeping sickness, Trypanosomes, Tsetse flies, Climate, Temporal variation, Bipindi, Cameroon

## Abstract

**Background:**

Human African Trypanosomiasis (HAT) remains a public health problem in many poor countries. Due to lack of financial resources in these countries, cost-effective strategies are needed for efficient control of this scourge, especially the tsetse vector. It was shown that perennial water sources maintain a favourable biotope for tsetse flies and thus the transmission dynamics of sleeping sickness. The present paper aimed at assessing the transmission dynamics of HAT in a forest environment where the hydrographic network is important.

**Methods:**

Two entomological surveys were carried out in July 2009 and March 2010 in the Bipindi sleeping sickness focus of the South Region of Cameroon. Entomological and parasitological data were collected during both trapping periods (including the climate variations throughout a year) and compared to each other. The level of risk for transmission of the disease during each trapping period was also evaluated at the trap level and materialised on the map of the Bipindi focus.

**Results:**

*Glossina palpalis palpalis* was the most prevalent tsetse fly species captured in this focus. The overall densities of tsetse flies as well as the risk for transmission of HAT in the Bipindi focus were significantly higher in July than in March. At the trap level, we observed that these parameters were almost constant, whatever the trapping period, when the biotope included perennial water sources.

**Conclusions:**

This study shows that the spatial distribution of traps, as well as the temporal climatic variations might influence entomological and parasitological parameters of HAT and that the presence of perennial water sources in biotopes would favour the development of tsetse flies and thus the transmission of sleeping sickness. These factors should, therefore, be taken into account in order to provide more efficient vector control.

## Background

Human African Trypanosomiasis (HAT), also known as sleeping sickness, remains a public health problem in Sub-Saharan Africa, where up to 30,000 cases are currently reported [1]. This vector-borne parasitic disease is due to a protozoan belonging to the genus *Trypanosoma*, transmitted to humans via bites of tsetse flies of the genus *Glossina*. Sleeping sickness transmission mostly relies on the vector competence, the host behaviour, and the ecology of tsetse flies [2,3]. The prevalence of HAT thus differs from one endemic country to another as well as in different parts (focus or village level) of a single country [1]. Up till now, diagnosis and treatment of infected people remains the cornerstone of sleeping sickness control programs in most endemic countries. This strategy led to the reduction of up to 68% of the number of new cases reported from 1995 to 2006 [4]. Following this success, elimination of the disease has become feasible. However, to achieve this elimination, it is important to take into consideration all factors involved in the epidemiology of sleeping sickness, notably the animal reservoir, which has been reported in the Gambian form of HAT and which could play an important role in the maintenance of the parasite after medical surveys. Although fighting against the animal reservoir is difficult to undertake, it has been shown that, after medical surveys, the trapping of tsetse flies enabled reduction of the prevalence of trypanosome pathogens to man in animals of the forest regions of Cameroon [5]. Vector control consequently appears to be an alternative method to tackle the animal reservoir. In the savannah zones, transmission generally occurs in a limited number of biotopes, such as water points and their vicinities where tsetse flies and mammals are frequently encountered, respectively for ecological reasons and water needs. Such biotopes could, therefore, be identified and prioritised during vector control campaigns [6,7]. However, the transmission pattern of the disease in the forest regions is quite different from that prevailing in savannah. The transmission could occur everywhere in the forest regions due to the hydrographic network, which favours the presence of tsetse flies and mammals in different types of biotopes. For cost-effective and sustainable vector control in the forest regions, there is a need to find appropriate strategies that can identify localised areas of the environment presenting higher transmission risk and where the vector control must be prioritised. To achieve this aim, it is important to identify and characterise the main factors involved in the transmission of the disease. The identification of trypanosomes in tsetse flies is an indication that the transmission of the parasite occurs in the region. Moreover, the identification of blood meals provides information on the nutritional behaviour of tsetse flies under natural conditions. These biological factors can be combined with environmental factors to evaluate the transmission risk index [8]. This indicates that the biological and environmental factors involved in the transmission of the disease are important while planning appropriate and efficient vector control.

In this study, entomological and parasitological data were generated from two entomological surveys in order to estimate the transmission risk index in each trapping site and in villages of the Bipindi sleeping sickness focus. Thereafter, the transmission risk indices were incorporated in the map of the Bipindi focus to identify zones presenting high risk for the disease transmission and where vector control must be prioritised to achieve the elimination goal in the forest regions of Sub-Saharan Africa.

## Methods

### Study area

The present study was conducted in the Bipindi (3°2’N; 10°22’E) sleeping sickness focus, located in the South Region of Cameroon, at approximately 75 km from the Atlantic Ocean. It has been known as “the Lolodorf focus” since the beginning of the 20^th^ century. This focus did not receive much attention (due to relatively low prevalence of the disease in this area) until 1998-1999 where 44 cases were diagnosed in two villages (Lambi and Bidjouka) of this region [9,10]. Thereafter, periodic medical surveys were conducted and about 83 cumulative cases were reported in this focus between 1998 and 2011 (Ebo’o-Eyenga, personal communication). This region has an equatorial climate divided into four seasons. Bipindi’s landscape is dominated by a dense evergreen forest made of bamboos, cocoa and coffee plantations. The hydrographic network is intricate with many streams and rivers that offer suitable habitats to tsetse flies. The activities of the Bipindi’s population are hunting, fishing, agriculture, extraction of palm wine and palm oil, and rearing of livestock. This study was performed in three villages (Lambi, Memel I and Ebiminbang) located on the three principal road axes of the focus.

### Trapping and dissection of tsetse flies

During each entomological survey, 12 pyramidal traps [11] were deployed for 9 (in July) or 8 (in March) consecutive days in each village. The geographical coordinates of each trap were recorded using a high sensitivity global positioning system [GPS eTrex^®^; Garmin (Europe) Ltd, Southampton, U.K.] (Table 1). Trapped tsetse flies were harvested once a day. They were identified by morphological criteria, counted and then sorted into teneral and non-teneral as previously described by Laveissière et al. [12]. All living non-teneral flies were dissected in a drop of 0.9% saline solution using a stereo-microscope. Midguts were examined under a light microscope (magnification x100) for the presence of trypanosomes and blood meals. The dissecting instruments were carefully cleaned after processing each fly to prevent cross-contamination. Examined midguts (with and without blood meal) were transferred into separate microfuge tubes containing 95° ethanol. These tubes were kept at room temperature in the field and stored at -20°C in the laboratory until use.

**Table 1:** Spatial distribution and seasonal variations observed in the environment of traps

**Trap ID**	**Latitude**	**Longitude**	**Seasonal variation observed in the environment of the trap**
**Lambi village**			
P101	N 3.10220^o^	E 10.44960°	None
P102	N 3.14750^o^	E 10.38920°	None
P103	N 3.10200^o^	E 10.44860°	None
P104	N 3.10020^o^	E 10.44780°	None
P105	N 3.10000^o^	E 10.44710°	None
P106	N 3.09970^o^	E 10.44640°	In March, a cultivated field was created near Mougue river, also used as bathing and laundry spot
P107	N 3.04470^o^	E 10.47640°	None
P108	N 3.09940^o^	E 10.44620°	None
P109	N 3.09870^o^	E 10.44330°	None
P110	N 3.09820^o^	E 10.44340°	None
P111	N 3.09510^o^	E 10.43950°	None
P112	N 3.09450^o^	E 10.43960°	None
**Memel I village**			
P113	N 3.14200^o^	E 10.38630°	None
P114	N 3.13910^o^	E 10.38700°	In March, pigs where replaced by sheep and goats in their enclosure
P115	N 3.09390^o^	E 10.43830°	None
P116	N 3.04470^o^	E 10.47460°	None
P117	N 3.09230^o^	E 10.43630°	None
P118	N 3.13660^o^	E 10.39160°	None
P119	N 3.13740^o^	E 10.39150°	None
P120	N 3.13700^o^	E 10.39070°	None
P121	N 3.14740^o^	E 10.38790°	None
P122	N 3.12700^o^	E 10.39360°	None
P123	N 3.14700^o^	E 10.38740°	None
P124	N 3.14720^o^	E 10.38580°	None
**Ebiminbang village**			
P125	N 3.04400^o^	E 10.47330°	The stream and a swampy area associated dried up in March
P126	N 3.12690^o^	E 10.39350°	The stream dried up in March and the field previously cultivated in July was empty
P127	N 3.04480^o^	E 10.47300°	A stream used as bathing and laundry spot dried up in March
P128	N 3.04760^o^	E 10.47520°	None
P129	N 3.14210^o^	E 10.38900°	None
P130	N 3.04370^o^	E 10.47160°	The swampy area in the bush dried up in March
P131	N 3.04610^o^	E 10.47040°	The stream and a swampy area associated dried up in March
P132	N 3.04680^o^	E 10.47520°	None
P133	N 3.04670^o^	E 10.47570°	None
P134	N 3.04700^o^	E 10.47500°	None
P135	N 3.04610^o^	E 10.47030°	The stream dried up in March and the field previously cultivated in July was empty
P136	N 3.04470^o^	E 10.47550°	The stream dried up in March

### DNA extraction procedure

Samples were thawed and incubated at 80°C for ethanol evaporation. Then, 300μl of 5% chelex was added to each tube containing the tsetse midgut and mixed by pulse-vortexing for 10 min [13]. The homogenised mixture was incubated at 56°C for 30 min and then at 98°C for 30 min. After centrifugation at 16,000g for 10 min, the supernatant (DNA extract) was collected and stored at -20°C for Polymerase Chain Reaction (PCR) analyses.

### Identification of trypanosome species

DNA amplification was performed as previously described [14], using primers specific for *Trypanozoon* (TBR1: 5’-GAATATTAAACAATGCGCAG-3’ and TBR2: 5’-CCATTTATTAGCTTTGTTGC-3’); *T. congolense “*forest type” (TCF1: 5’- GGACACGCCAGAAGTACTT-3’ and TCF2: 5’-GTTCTCGCACCAAATCCAAC-3’); *T. congolense “*savannah type” (TCS1: 5’-TCGAGCGAGAACGGGCACTTTGCGA-3’ and TCS2: 5’-ACAATTAGGGACAAACAAATCCCGC-3’); and *T. vivax* (TVW1: 5’-CTGAGTGCTCCATGTGCCAC-3’ and TVW2: 5’-CCACCAGAACACCAACCTGA-3’) [15]. PCR products were resolved on 2% agarose gels stained with ethidium bromide (0.3 μg/ml). For *Trypanozoon* positive samples, another PCR was carried out as described by Herder et al. [16] using TRBPA1/2 primers (TRBPA1: 5’-GCGCCGACGATACCAATGC-3’ and TRBPA2: 5’-AACGGATTTCAGCGTTGCAG-3’), which amplify an allele of 149 bp characteristic of *T. b. gambiense* group 1. The amplification products were resolved on 10% polyacrylamide gels. The electrophoresis was performed in 1X TBE buffer at 120V and 25 mA during 15 hrs. After staining in a solution containing ethidium bromide, the gel was washed in 1X TBE buffer and visualised under UV light.

### Identification of the origin of tsetse fly blood meals

The origin of tsetse fly blood meals was determined using the modified heteroduplex PCR-based assay as described by Njiokou and colleagues [17]. Briefly, Cytochrome B gene was amplified in 25μl of a mixture containing 10mM Tris–HCl buffer (pH 9), 50mM KCl, 1.5mM MgCl_2_, 16 pmol of each of the reverse and forward vertebrate Cytochrome B primers (CYTB1: 5’-CCCCTCAGAATGATATTTGTCCTCA-3’ and CYTB2: 5’-CCATCCAACATCTCAGCATGATGAAA-3’), 200μM of dNTPs, 0.4 units of Taq DNA polymerase and 2.5μl of the template (DNA extract or distilled water for negative control). The PCR reactions consisted of a denaturation step at 95°C for 3.5 min and 40 amplification cycles. Each cycle included a denaturation step at 95°C for 30s, an annealing step at 58°C for 50s and an extension step at 72°C for 1min. A final extension was performed at 72°C for 5min [18]. From the amplified fragments, heteroduplexes were formed by hybridization with Giant rat (*Crycetomys gambianus*) Cytochrome B DNA chosen as a driver [17]. The heteroduplex DNA profiles were resolved on 5% acrylamide/urea gels. The blood meal origin was identified by comparing the heteroduplex profile of each blood meal with those of vertebrates, including man, pig, goat and sheep, used as references.

### Data analysis

In the present study, traps were set up at the same positions during the two entomological surveys. For more accurate information on the conditions prevailing during these two trapping periods of survey, data on climate (temperature and rainfall) of the region of Bipindi were collected from the Cameroon National Meteorological Service, and an ombrothermic diagram drawn [see Additional file 1: Figure S1].

The apparent density per trap per day (ADT) was used to assess the relative abundance of tsetse flies during each survey and in each type of biotope.

$$\mathrm{ADT}=\frac{C}{\mathrm{TD}},$$

where *C* is the number of flies caught, *T* the number of traps deployed and *D* the number of days of trapping.

The risk for transmission of sleeping sickness was evaluated using the simplified entomological index (r), which is proportional to the apparent density of tenerals and the frequency of contact between humans and tsetse flies [2,7,19].

$$r=10^{4}{\left(t+1\right)}^{1.23}.n^{2}.\frac{C^{0.46}}{{\left(\mathrm{TD}\right)}^{3.69}}$$

In this formula, *C* represents the number of flies caught, *T* the number of traps deployed, *D* the number of days of trapping, *t* the number of teneral flies, *n* the number of human blood meals. To visualise the risk for the transmission of sleeping sickness on the map of the Bipindi focus, a thematic analysis was performed using the MapInfo Professional software version 8.5. The analysis was carried out at the trap level and for the two entomological survey periods in order to evaluate the spatial and temporal variation of the TRI.

Statistical analyses were undertaken to compare entomological and parasitological data between the two surveys according to the trapping period (including the climatic variations). Student’s *t*-test for paired-samples (traps were set at the same position during both surveys) was used to compare the ADT between trapping periods. The percentage of trapped and teneral flies, the trypanosome infection rates as well as the proportions of blood meals were compared between the two surveys by Chi-squared test. For small sample size, Yates correction for continuity or Fisher's exact test was used.

Student’s t-tests as well as Chi-squared tests were performed online using VassarStats computational website [20]. The threshold for significance was set at 5%.

## Results

### Entomological surveys

**Figure 1:**
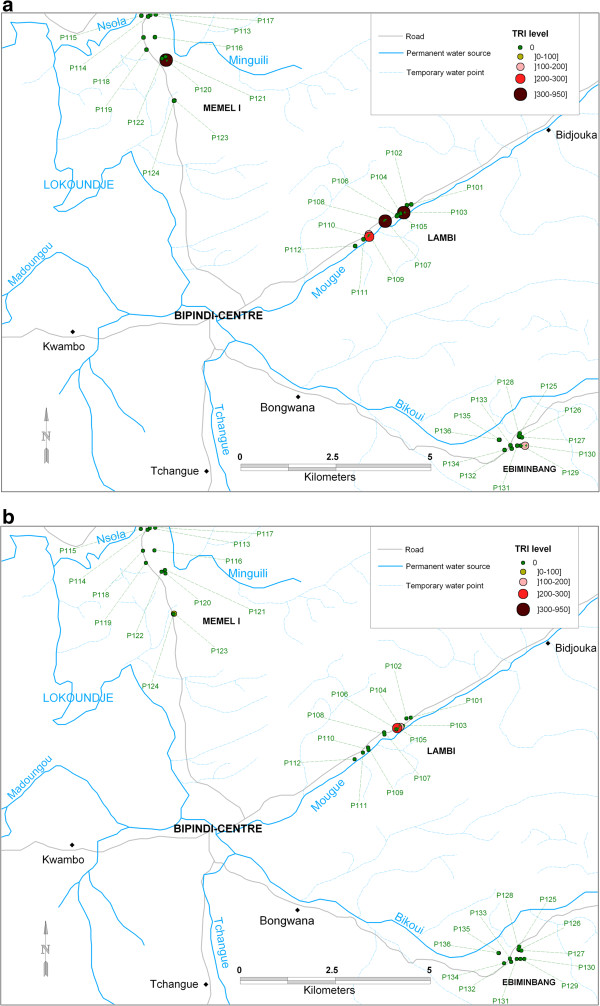
**Spatial distribution of traps and the associated transmission risk indices in July (a) and in March (b).** P101-P136 represents the trap ID.

During the two entomological surveys, 72 traps were set up and 1421 tsetse flies were caught: 1010 in July and 411 in March (Table 2). The number of flies caught, either in July or in March, was higher in traps set near permanent water sources (Figure 1a and 1b), especially at points where some activities associated with the presence of water (swimming or bathing, laundry and fishing) were generated. A significant difference was found between the proportion of flies trapped in July (71.1%) and in March (28.9%) (*χ*^2^ = 505, *p* < 0.0001).

**Table 2:** Results of the entomological surveys and parasitological examinations according to each trap

**Trap ID**	**Trapped**		**ADT**		**Teneral**		**Dissected**		**Blood meals**		**TV**		**TCF**		**TCS**		**Tbsl**		**Tbg**	
	**J**	**M**	**J**	**M**	**J**	**M**	**J**	**M**	**J**	**M**	**J**	**M**	**J**	**M**	**J**	**M**	**J**	**M**	**J**	**M**
**Lambi village**																				
P101	17	5	1.89	0.63	6	0	11	5	0	0	0	0	0	0	0	0	2	0	0	0
P102	10	7	1.11	0.88	3	0	7	4	0	0	0	0	0	1	0	0	0	2	0	1
P103	98	11	10.89	1.38	18	0	80	7	1HB, 2UN	0	1	0	1	0	0	0	2	0	1	0
P104	124	46	13.78	5.75	29	3	95	37	1PB, 4UN	1HB	2	0	0	1	0	1	2	1	0	0
P105	40	42	4.44	5.25	9	5	31	23	0	1HB	0	0	1	0	0	0	1	2	1	0
P106	70	9	7.78	1.13	14	2	56	6	3UN	0	1	0	0	0	1	1	0	0	0	0
P107	21	36	2.33	4.50	2	5	19	23	0	0	1	0	0	0	0	0	0	1	0	0
P108	55	15	6.11	1.88	12	3	43	9	1HB	0	2	0	0	0	0	0	0	0	0	0
P109	21	12	2.33	1.50	7	3	14	6	1HB	0	3	0	0	0	0	0	0	2	0	0
P110	41	10	4.56	1.25	9	1	32	6	1HB	0	2	0	0	0	0	0	0	0	0	0
P111	2	18	0.22	2.25	0	2	2	8	0	0	0	0	0	1	0	0	0	1	0	0
P112	18	7	2.00	0.88	4	0	14	6	1UN	1PB	0	0	0	0	0	0	0	0	0	0
**Memel I village**																				
P113	10	11	1.11	1.38	3	2	7	6	0	0	1	0	0	0	0	0	0	0	0	0
P114	1	5	0.11	0.63	0	0	1	4	1PB	0	0	0	0	0	0	1	0	0	0	0
P115	2	2	0.22	0.25	0	0	2	2	0	0	0	0	0	0	0	0	0	0	0	0
P116	3	0	0.33	0.00	0	0	3	0	0	0	0	0	0	0	0	0	0	0	0	0
P117	3	8	0.33	1.00	0	2	3	5	0	0	0	1	0	0	0	0	0	0	0	0
P118	9	3	1.00	0.38	1	1	8	2	0	0	0	0	0	0	0	0	0	0	0	0
P119	11	2	1.22	0.25	1	1	10	1	0	0	1	0	0	0	0	0	0	0	0	0
P120	37	37	4.11	4.63	8	6	29	27	2HB, 2UN	1UN	0	1	0	1	0	0	1	6	0	0
P121	45	12	5.00	1.50	5	1	40	11	0	1UN	2	0	0	0	0	0	1	1	1	1
P122	18	6	2.00	0.75	5	1	13	4	0		1	0	0	0	0	0	0	1	0	0
P123	38	12	4.22	1.50	4	1	34	11	0	1HB	0	0	0	0	1	0	1	2	0	0
P124	23	8	2.56	1.00	1	0	22	6	1PB	0	0	0	0	0	1	0	1	0	0	0
**Ebiminbang village**																				
P125	71	14	8.88	1.75	16	3	55	8	1PB, 1UN	0	1	0	0	0	0	1	6	0	0	0
P126	22	6	2.75	0.75	5	2	17	1	1UN	0	0	0	1	0	1	0	0	0	0	0
P127	28	7	3.50	0.88	6	2	22	5	0	0	1	0	0	1	1	0	0	0	1	0
P128	9	0	1.13	0.00	4	0	5	0	1UN	0	0	0	0	0	0	0	0	0	0	0
P129	23	21	2.88	2.63	4	1	19	14	0	0	2	0	0	1	0	0	0	0	0	0
P130	33	11	4.13	1.38	5	2	28	5	1HB, 2UN	0	4	0	0	1	0	0	0	0	0	0
P131	5	5	0.63	0.63	0	0	5	4	0	0	0	0	0	0	0	0	0	0	0	0
P132	4	4	0.50	0.50	0	0	4	4	0	0	0	0	0	0	0	0	0	0	0	0
P133	15	4	1.88	0.50	3	1	12	2	1UN	0	0	0	0	0	0	0	0	0	0	0
P134	20	5	2.50	0.63	4	0	16	4	0	0	0	0	0	0	0	0	0	0	0	0
P135	31	5	3.88	0.63	8	1	23	3	0	0	2	0	2	0	1	0	6	0	0	0
P136	32	5	4.00	0.63	6	0	26	5	1UN	0	2	0	2	0	1	1	0	0	0	0
**Total**	**1010**	**411**	**3.23**	**1.43**	**202**	**51**	**808**	**274**	**7HB, 4PB, 19UN**	**3HB, 1PB, 2UN**	**29**	**2**	**7**	**7**	**7**	**5**	**23**	**19**	**5**	**2**

HB: Blood meals taken on humans; PB: Blood meals taken on pigs; UN: blood meals not identified despite the amplification of Cytochrome B gene; ADT: apparent density of tsetse flies per trap per day; Tbsl: *Trypanosoma brucei* s.l.; Tbg: *Trypanosoma brucei gambiense*; TCF: *Trypanosoma congolense* forest type; TCS: *Trypanosoma congolense* savannah type; TV: *Trypanosoma vivax*; J: June; M: March.

Three different tsetse fly species including *Glossina palpalis palpalis, G. pallicera* and *G. nigrofusca* were captured during both trapping periods; *G. p. palpalis* was the most predominant species (1002 (99.2%) in July and 406 (98.8%) in March), followed by *G. pallicera* (6 (0.6%) in July and 3 (0.7%) in March) and *G. nigrofusca* (2 (0.2%) in July and 2 (0.5%) in March). Taking each tsetse species alone, the proportion of flies caught was similar in both trapping periods (*p* > 0.05). The overall ADT was significantly higher in July compared to its value in March (t = 4.06, *p* < 0.0001).

Out of the 253 teneral flies caught in this study, 202 were collected in July whereas only 51 tenerals were captured in March. The proportion of teneral flies was significantly higher in July (*χ*^2^ = 11.5, *p* = 0.0007).

Of the 1082 dissected tsetse flies, 88 (64 in July and 24 in March) showed residual blood meals in their midguts. The proportion of the flies with blood meals was not significantly different (*p* > 0.63) between July (7.9%) and March (8.8%) (Table 2).

### Parasitological results and blood meal identification

Of the 1082 dissected flies, microscopy revealed 28 trypanosome infections; the infection rates were significantly higher in March (2.6%) compared to its value in July (0.7%) (*χ*^2^ = 4.24, *p* = 0.035).

PCR allowed the identification of 8.2% (71/808) and 12.8% (35/274) of trypanosome infections in July and in March respectively. Comparing the infection rates for each trypanosome species, no significant difference was found between the two trapping periods for *T. congolense* “forest type” (*χ*^2^ = 3.34, *p* = 0.067), *T. congolense* “savannah type” (*χ*^2^ = 0.95, *p* = 0.33) and *T. b. gambiense* (*p* = 1). However, the infection rates were significantly higher in March for *T. vivax* (*χ*^2^ = 6.01, *p* = 0.01) and *T. brucei* s.l. (*χ*^2^ = 9.16, *p* = 0.003) (Table 2).

Of the 88 midguts (64 in July and 24 in March) containing residual blood meals, the Cytochrome B gene was successfully amplified only for 36 (30 in July and 6 in March). The heteroduplex PCR method, processed only for successfully amplified Cytochrome B gene blood meals, revealed that 10 (7 in July and 3 in March) were from man and 5 (4 in July and 1 in March) from pig, the 21 (19 in July and 2 in March) remaining being unidentified (Table 2). No significant difference was found between the trapping periods for blood meals taken on man as well as on pig (*p* > 0.32).

### Risk for transmission of sleeping sickness

Figure 1a and 1b illustrate the transmission risk indices according to traps and villages. The risk for the transmission of sleeping sickness varies between traps of the same village and between villages of the same sleeping sickness focus. The transmission risk index (TRI) was higher in July (r = 3.26) than in March (r = 0.16) (Figure 1a and 1b). The Lambi village showed the highest TRI in both trapping periods (r = 30.10 in July; r = 1.21 in March). The TRI was higher in Memel I than in Ebiminbang both in July (0.87 and 0.70 respectively) and in March (0.13 and 0.00 respectively).

## Discussion

The primary goal of this study was to evaluate and map the transmission patterns of sleeping sickness prevailing in the Bipindi HAT focus during two contrasting periods in order to provide knowledge that will help to develop a cost-effective control strategy. Within this scope, entomological and parasitological parameters were recorded and compared in three villages of the Bipindi sleeping sickness focus.

During the entomological surveys, *G. p. palpalis* was the most trapped tsetse species. A significantly higher density (ADT) of tsetse flies was found in July but the highest infection rates were observed in March. Among the 15 successfully identified blood meals, 10 (66.7%) originated from humans and 5 (33.3%) from pigs. The highest transmission risk index was observed in the Lambi village; the evaluation of the TRI at the trap level shows that the risk was higher in July and near perennial water sources.

The presence of G*. p. palpalis*, *G. pallicera* and *G. nigrofusca* in the Bipindi sleeping sickness focus corroborates previous observations in the same focus [3,21]. In this study as well as in other studies carried out in the Bipindi sleeping sickness focus [19,22,23], the most prevalent tsetse species was *G. p. palpalis* (> 98.0%). Previous studies [19] using the “Vavoua” tsetse fly trap, which has been proven to be very efficient on a large variety of tsetse fly species [24], also revealed a high proportion of *G. p. palpalis* in the same environment where pyramidal traps were set up. These results show that *G. p. palpalis* was the most trapped tsetse species whatever the type of trap used in most sleeping sickness foci of the forest region of Cameroon. They also show that the higher proportion of *G. p. palpalis* observed in the present study cannot be a consequence of the trapping technique or to the type of trap used. Although this species has a high power of adaptation, the high prevalence observed is a reflection of the effect of human action on the biotopes where the traps were set [12,21].

The significantly higher ADT observed in July corroborates results obtained in previous investigations in the Bipindi focus [19] as well as in the Campo sleeping sickness focus in the forest region of the southern Cameroon [22]. The relative abundance of tsetse flies showed high correlation with factors such as climate, vegetation or the contact frequency between tsetse flies and their vertebrate hosts [19]. It was also shown that the type of soil, human activities, the breeding sites of tsetse flies as well as the distribution of mammals might significantly influence the abundance of tsetse flies in a given biotope [25,26]. In our study, the traps were set in the same position during both trapping periods to allow the measurement of the direct or indirect impact of climate (temperatures and rainfalls) variations on the abundance of flies at the same capture sites. Therefore, the changes occurring in the environment or in the host behaviour throughout the trapping period are driven by the temporal variations of the climate. Indeed, the climatic conditions prevailing in July (beginning of the little dry season) and March (end of the major dry season) were probably highly influenced by the cumulative effect of the climatic conditions prevailing during the months or seasons preceding each trapping period, as shown in the ombrothermic diagram [see Additional file 1: Figure S1]. Subsequently, some water sources tended to dry up after the dry months and to be regenerated after rains. However, permanent water sources were maintained throughout the year, thus favouring the development and maintenance of a micro-habitat for tsetse flies. The significant drop in ADT (from 3.05 in July to 0.91 in March) in Ebiminbang may result from the environment around each trap. In fact, most streams in this village are not permanent during the year. Therefore, almost all traps in Ebiminbang were set up around temporary water sources that dried up in March (Table 1, Figure 1a and 1b). On the contrary, in Memel I and Lambi, most of the traps were set across the Miguili and Mougue streams (these permanent streams are the tributaries of the Lokoundje river) respectively. A noteworthy observation in this study is that fly densities were particularly elevated where host activities, in and/or around large and perennial water sources, were generated. Consequently, the ADT were almost constant or not significantly affected from one trapping period to another. These results confirm clearly that the water availability, along with the associated activities of hosts (bathing, swimming, laundry), may have a real impact on tsetse fly density and consequently on the transmission of sleeping sickness.

The significantly higher proportion of teneral flies observed in July compared to the value in March might be due to some environmental conditions (climate, vegetation …), which favour the completion of the fly life-cycle [12]. During the dry months, the high temperature does not favour the development of pupae because dry soil does not enable tsetse flies to put their larvae in appropriate conditions or in environments where they can easily develop. Ideally, precipitation should be moderate, an excess of rain can drain the soil and lead to the destruction of pupae buried by tsetse flies [27]. As expected, the little rainy season preceding the sampling in July was favourable to the development of tsetse flies. Furthermore, the higher proportion of teneral flies recorded in July reflects the higher transmission risk index observed during this trapping period because teneral flies are highly susceptible to infections during their first blood meal [12].

The higher proportion of tsetse flies harbouring a blood meal in March compared to July could also be explained by the climatic conditions. Though tsetse flies can find favourable climatic conditions and diverse hosts everywhere in the forest zone, the temperature in the dry period is high and vertebrate hosts move close to permanent water sources and shade where they can get water or rest. In such biotopes, tsetse flies can easily get their blood meals [12]; this is in accordance with the higher proportion of blood meals observed in March. Despite the identification of human and pig blood meals in tsetse flies of the Bipindi sleeping sickness focus, most blood meals were not identified. These unidentified blood meals were probably from wild animals because the most common domestic animals found in this focus were used as a reference during the identification of blood meals [28]. This hypothesis is strengthened by previous results in the Bipindi HAT focus where tsetse blood meals originating from different wild animal species including *Python sebae*, *Tragelaphus spekeii*, *Trionyx* and *Kinixys* were reported [3,29].

The high risk for HAT transmission observed in July (Figure 1a and 1b) is not in line with results obtained by Grébaut and colleagues [19] who found a similar level of the transmission risk during the dry and the rainy seasons. The transmission risk indices found by these authors were 2.03 in May (rainy season) and 2.09 in February (dry season), despite the difference in the relative density of tsetse flies during the two periods (2.3 in May and 1.7 in February). The discrepancy between our results and those of Grébaut et al. [19] could be explained by the sampling period. Grébaut et al. [19] sampled tsetse flies in May (in the middle of the little rainy season) and in February (at the end of the major dry season), whereas our surveys were conducted at the beginning of the little dry season and at the end of the major dry season. Another explanation of this discrepancy will be the variation in the rainfall over the years as observed in March [see Additional file 1: Figure S1]. The comparison of the transmission risk indices between villages shows that the Lambi village had the highest level of the transmission risk, whatever the trapping period. This result is in line with those obtained during medical surveys where most of the patients previously diagnosed were from this village. The high transmission risk index observed in Lambi could also be explained by some environmental factors such as permanent water sources and stable vegetation cover which favour a high level of sleeping sickness transmission. In Ebiminbang, however, most of the rivers dried up during the dry seasons. This may explain the drop of the ADT as well as the low value of the transmission risk index observed in this village.

## Conclusions

The results of this study showed the impact of spatial and temporal climatic variations on the entomological and parasitological parameters characterising the transmission of sleeping sickness in the Bipindi focus. They also showed that the density of tsetse flies as well as the sleeping sickness transmission risk can be influenced by the conditions prevailing before the trapping period. The water availability (permanent or temporary) is an important component of the environment which has a great influence on the vegetation cover, the fly density, and hence on the transmission of sleeping sickness. To foresee effective vector control in the sleeping sickness foci, especially in forest zones, it appears important to better understand the spatial and temporal climatic variations occurring in each focus since such variations may have real impacts on the transmission of the sleeping sickness.

## Competing interests

The authors declare that they have no competing interests.

## Authors’ contributions

JTL collected field data, carried out molecular biology experiments and drafted the manuscript. HND collected field data, performed the statistical analyses and drafted the manuscript. GS participated in the conception of the study and in the design of experiments, collected field data and helped to draft the manuscript. GRN collected field data and helped to draft the manuscript. GC participated in the conception of the study and helped to draft the manuscript. TA participated in the conception of the study and helped to draft the manuscript. FN participated in the conception and the coordination of the study, designed the experiments, collected field data and helped to draft the manuscript. All authors read and approved the final manuscript.

## Supplementary Material

Additional file 1: Figure S1 Ombrothermic diagram of the years of the entomological surveys in the Bipindi sleeping sickness focus. The dark bars indicate months during which the surveys took place. Click here for file
