# Supplementary figures and images for: Spatial and temporal variations relevant to tsetse control in the Bipindi focus of southern Cameroon
Source: Parasit Vectors. 2013 Jul 1;6:193. doi: 10.1186/1756-3305-6-193 (PMC3701558; doi:10.1186/1756-3305-6-193)

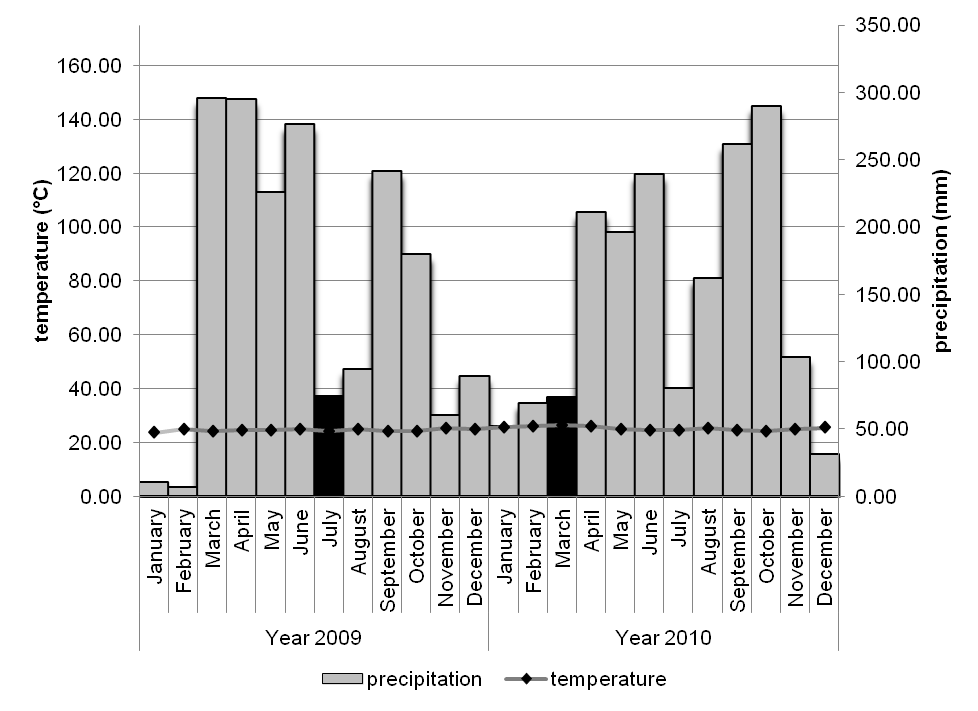

Supplement: Additional file 1: Figure S1 — Ombrothermic diagram of the years of the entomological surveys in the Bipindi sleeping sickness focus. The dark bars indicate months during which the surveys took place. [file 1756-3305-6-193-S1.tiff]
